# Engaging parents in digital sexual and reproductive health education: evidence from the JACK trial

**DOI:** 10.1186/s12978-020-00975-y

**Published:** 2020-08-27

**Authors:** Áine Aventin, Aisling Gough, Theresa McShane, Kathryn Gillespie, Liam O’Hare, Honor Young, Ruth Lewis, Emily Warren, Kelly Buckley, Maria Lohan

**Affiliations:** 1 School of Nursing & Midwifery and Centre for Evidence and Social Innovation, Queen’s University Belfast, Belfast, Northern Ireland, UK; 2 School of Social Sciences, Education and Social Work and Centre for Evidence and Social Innovation, Queen’s University Belfast, Belfast, Northern Ireland, UK; 3 School of Social Sciences, Cardiff University, Cardiff, Wales, UK; 4 MRC/CSO Social and Public Health Sciences Unit, Univeristy of Glasgow, Glasgow, Scotland, UK; 5 Department of Public Health Environments and Society, London School of Hygiene and Tropical Medicine, London, England, UK

**Keywords:** Sexual and reproductive health, Sex education, Relationships and sexuality education, Digital health, Parent engagement, Adolescent health, Interventions, Randomised controlled trial, Process evaluation

## Abstract

**Background:**

Research evidence and international policy highlight the central role that parents play in promoting positive sexual behaviour and outcomes in their children, however they can be difficult to engage in sexual and reproductive health (SRH) education programmes. Digital health promotion that uses online and mobile technologies (OMTs) to promote parent-child communication may offer an innovative solution to reach parents, however, few programmes have used OMTs to involve parents in SRH, and none have reported lessons learned in relation to optimising engagement. This study addresses this gap in the literature by reporting acceptability and feasibility of using OMTs to engage parents in SRH education. Findings will be relevant for those wishing to develop and implement digital SRH programmes with parents internationally.

**Methods:**

The *Jack Trial* is a UK-wide cluster randomised controlled trial recruiting over 8000 adolescents from 66 socially and religiously diverse post-primary schools. An embedded mixed-methods process evaluation explored user engagement with parent components of the *If I Were Jack* SRH education programme, which include online animated films and a parent-teen homework exercise.

**Results:**

A total of 109 adolescents, teachers, parents and SRH policy experts took part in semi-structured interviews and focus groups, 134 parents responded to an online survey, and 3179 adolescents completed a programme engagement and satisfaction questionnaire. Parents who accessed the materials were positive about them; 87% rated them as ‘good or excellent’ and 67% said they helped them have conversations with their child about SRH. Web analytics revealed that 27% of contacted parents accessed the digital materials, with 9% viewing the animated films. Only 38% of teachers implemented the homework exercise, mainly because they assumed that students would not complete it or it might result in backlash from parents.

**Conclusions:**

While digital parental materials show promise for engaging parents in SRH education, this study suggests that in order to optimise engagement, parental components that give parents the necessary skills to have conversations with their children about sex should be coupled with efforts to increase school and teacher confidence to communicate with parents on sensitive topics.

**Trial registration:**

ISRCTN99459996.

## Plain English Summary

We now understand that successful school-based Relationships and Sexuality Education (RSE) programmes need to take a multi-pronged approach involving adolescents, peers, parents, and wider community-based service providers [1, 2]. Internationally, schools are therefore increasingly encouraged to involve parents in RSE, yet few school-based RSE programmes manage to do this successfully. Barriers to engaging parents include parents’ inability to attend workshops due to other commitments, lack of time or motivation, perceived embarrassment or underestimations of the value of RSE [3]. Digital health promotion that uses online and mobile technologies (OMTs) to promote parent-child communication may offer an innovative solution to reach parents [4]. However, there are few existing programmes that use OMTs and insufficient evidence regarding the acceptability of these methods in general. To better understand how we might engage parents and encourage communication with their children about sexual and reproductive health (SRH), we worked with UK parents, teachers and SRH experts to make two animated films, which were delivered as part of an RSE programme during a UK-wide school-based research trial. The aim of the study was to assess user engagement with the parental components in relation to three concepts: 1) implementation fidelity (was this programme component carried out as intended?); 2) acceptability and feasibility (if carried out, was it considered good or bad?); and 3) general barriers and facilitators to using OMTs with parents in school-based SRH promotion. The findings offer recommendations for programme development and future research seeking to use digital SRH education for parents. A key message is that while digital parental materials show promise for engaging parents in SRH education, in order to optimise engagement, parent materials that address barriers to parental engagement should be coupled with efforts to increase school and teacher confidence to communicate with parents on sensitive topics.

## Background

### Adolescent sexual risk behaviour

Sexual initiation and activity in adolescence is common. While statistics vary greatly by country, gender, age, ethnicity and socio-economic status [5–11], in most countries around the world at least a third of unmarried adolescents have had sexual intercourse by the time they are 19, with some form of partnered intimate activity common among 14-year-olds [7, 12–19]. Sexual risk behaviours such as early sexual initiation, sex with multiple partners, and inconsistent contraceptive use are associated with unintended early pregnancy and increased susceptibility to sexually transmitted infections (STIs) including HIV [9, 18, 20]. In high-income countries such as the UK, around half of teenage pregnancies result in abortion; for those teenagers who do become parents, negative health, economic and social outcomes are reported [21]. Further, infant mortality rates are 60% higher for babies born to teenage mothers and children of teenage mothers are more likely to experience prematurity, low-birth weight, poverty and to become teenage parents themselves [21].

### Parental influences on adolescent risk behaviours

Parents and primary caregivers play a central role in adolescents’ lives and research demonstrates that they can influence their children’s sexual behaviours including condom use and the timing of, and circumstances surrounding, sexual initiation [1, 22–32]. Often indicated as adolescents’ primary source of information about contraceptive decision-making [33], parents can play an important role in supervising adolescent activity, conveying appropriate sexual health information to their children, modelling open and respectful communication about sex, and can exert a substantial influence on adolescents’ attitudes, values and beliefs in relation to SRH [26–28, 30]. Meta-analyses have demonstrated that SRH programmes involving parents improve communication about SRH between parents and adolescents [29] and increase safer sex behaviours [23, 30].

### Parent-teen communication about sex

Despite the potential for positive effects and the fact that both parents and adolescents report wanting to communicate about SRH [34–37], a significant proportion of adolescents around the world report rarely or never discussing sex with their parents [30, 32, 35, 38–41]. Parents often fail to have timely discussions, with as many as 40% of adolescents engaging in sexual behaviour before parents discuss SRH with them [42]. Barriers such as embarrassment, inaccurate knowledge, low self-efficacy, religious and cultural beliefs opposed to comprehensive RSE, and parental underestimation of their child’s sexual behaviour, may prevent some parents from communicating about these issues [34, 35, 43–45]. In one study parents reported that while during pre-adolescence their children often initiated discussions with them about sex and sexuality, by the teenage years, adolescents were more inclined to close down such conversations initiated by parents [41]. Further, lack of communication at home is sometimes coupled with a lack of RSE at school when parents exercise their right to remove their children from school-based RSE. While research suggests that most parents want their children to obtain accurate and comprehensive RSE in schools, some parents are fearful that it will undermine ethno-religious beliefs. This perceived clash of values has recently led to high-profile public demonstrations of parents outside schools in the UK [46]. Specifically, these parents are protesting against the UK Government’s plans in England and Wales to disallow parental rights to withdraw their child from newly introduced government mandated RSE education [47, 48].

Adolescents also report mixed views about engaging in SRH-related activities with their parents [49, 50] with feelings of awkwardness, generational differences and relationship difficulties constituting key barriers. The perceived implications of conversations about SRH is also key, with many young people hesitant about initiating conversations that might lead their parents to assume they are having sex [51, 52] and some parents fearing that discussing SRH may encourage sexual activity [45].

### Gender differences in intergenerational communication about sex

Internationally, mothers feature more predominantly than fathers in relation to communicating with their children about sex, particularly in relation to communicating with daughters [1, 30, 53–55]. In a study of UK adolescents [54], 43% of girls reported that their mother was a source of information about sex, while only 7% cited their father. Among boys, a similar proportion cited either their mother (20%) or father (18%). Further, it appears that young people themselves (particularly girls) have a preference for same-sex communication with parents [54, 55]. In Tanton et al.’s study [54] of adolescents who felt they ought to have known more about sex, 40% of girls reported their mothers as a preferred source, while 23% of boys cited their fathers and 15% cited their mothers.

While there has been little research exploring fathers’ roles as sex educators, it has been suggested that whilst they often aspire to be able to speak to their children about sex in order to promote father-child intimacy, they are hindered by gendered norms that place mothers in the role of primary parental sex educators for both sons and daughters [41, 56]. Further, father’s silence in relation to communicating has been related to fathers’ constructions of ‘sexuality as taboo’; perceptions that are thought to endure from their own experiences of sex education as children [57].

Research also suggests that gender differences exist in relation to the *impact* of parent-child communication about sex on sexual behaviour and outcomes, with impacts emerging as more significant following mother–child than father–child communication [30]. A recent study exploring father-son communication among African Americans, however, demonstrated that father–son communication is an important factor in decreasing adolescent males’ sexual risk behaviours and HIV risk [58].

### Parent-teen communication programmes

While increasingly, school-based RSE programmes attempt to address these barriers [29], the provision and evaluation of educational and pragmatic tools for parents has been largely absent. In a recent meta-analysis of trials seeking to reduce STI risk among adolescents, only 12.7% involved parents as programme participants [59]. This overlooks evidence indicating that programmes that reach beyond the classroom (including those with parental, peer and community components) are more effective [60–63], particularly with adolescent men [64]. RSE programmes that do not engage parents ignore the fact that adolescents influence, and are influenced by, the world around them [65, 66]. Indeed, it is well-recognised that a ‘whole school approach’ (which involves young people, school staff, parents and the wider community in the development of RSE curricula) is one of the most important elements of effective RSE [47, 67]. This approach has been embedded within the development of the new curriculum in Wales [67],recently proposed as a positive approach by the Department of Education in England [47] and recommended by international policy relating to sexual education [4].

The neglect to involve parents in RSE may be, in part, explained by reported difficulties engaging them [3, 68] and the expense of facilitated face-to-face workshops, which most programmes with parents to date have utilised [29]. The JACK feasibility trial engaged only 7% of eligible parents in its school-delivered face-to-face workshops [3, 52] and other research reports similar difficulties engaging parents [69–72]. Barriers to engagement and implementation fidelity (although underreported in the literature) are likely to include parents’ inability to attend workshops due to other commitments, lack of time or motivation, perceived embarrassment or underestimations of the value of RSE in general [3]. There have, therefore, been calls for innovative programmes that can help extend reach, while also providing parents with knowledge about SRH and guidance for successful communication with adolescents [29, 35, 43].

### Digital RSE

Online and mobile technologies (OMTs) may present opportunities for increasing reach and decreasing perceived embarrassment of parental involvement in RSE. An advantage of OMTs is that they offer the potential for providing innovative, evidence-informed technologies that increase reach and help maintain implementation fidelity [50]. Further, low-dose, self-directed, easily-disseminated modes of delivery, have been found to have similar effects than high-dose, intensive (and much more expensive) programmes [29, 34, 73].

While the potential of these modalities has been highlighted [50, 74], there remain a lack of programmes utilising OMTs to engage parents and a dearth of research evidence regarding implementation fidelity and end-user perceptions of their acceptability and feasibility. In a 2013 review of 44 programmes that involved parents in SRH education [1], none made use of OMTs for behaviour change. By 2019, a review of 31 SRH programmes involving parents, included only two that made use of digital methods [23], with one of these reporting the effects of mass media (TV and radio) messages rather than OMT components [75]. We located only three studies that used OMTs to engage parents in SRH education [76–78]. While all three report positive impacts on parent-teen communication, none consider implementation, and all offered monetary incentives for participation, making it difficult to determine the external validity of the programmes.

These limitations impose barriers to understanding the real-world effectiveness and reach of existing programmes and also point to a gap in knowledge relating to our understanding of the acceptability and feasibility of digital programmes for engaging parents. This study addressed this gap by working with UK parents, teachers and RSE policy experts to co-produce digital parental components for an RSE programme and implementing them during a UK-wide school-based cluster randomised trial [79]. The aim was to assess implementation fidelity of parental digital components; determine their acceptability and feasibility; and explore barriers and facilitators of using digital programmes as a means of engaging parents in school-based SRH promotion. Drawing on the study’s innovative focus on boys (as well as girls) and inclusion of faith-based schools in its sample, we also consider the implications of the findings for using digital methods to promote parent-son communication and facilitate SRH education in faith-based schools, issues that are of utmost contemporary importance.

## Methods

### Intervention

*If I Were Jack* [52, 79, 80] is an evidence-informed, theory-based, gender-transformative (challenges gender inequalities relating to SRH) RSE programme designed to reduce unintended teenage pregnancy and promote positive sexual health. It aims to increase intentions to avoid teenage pregnancy by encouraging delayed initiation of sexual intercourse and/or consistent use of contraception and is designed to be delivered in educational settings. It specifically targets boys aged 14–15, however, it can also be delivered to girls and used in same-sex or mixed-class groups. It is designed to promote critical thinking about the social pressures that normally situate teenage pregnancy and its prevention as a female-only issue. Programme components include: an interactive film which tells the story of 16-year-old Jack, who has just found out that his girlfriend Emma is unexpectedly pregnant; classroom materials for teachers containing detailed lesson plans with specific classroom-based and homework activities; a 90-min training session delivered by RSE specialists to teachers implementing the programme and parent components as described below. The JACK programme and Trial methods are described in full elsewhere [52, 79] and more information about the project can found at https://www.ifiwerejack.com.

#### Parent components

One element of the *If I were Jack* theory of change involves increasing self-efficacy in communicating about SRH among parents/carers and teens. This is built into the programme in two ways. First, the programme includes an optional homework exercise that invites students to ‘interview’ their parents/primary caregivers (or another trusted adult such as an older sibling or relative) at home, about their thoughts on Jack and Emma’s situation, after they have watched an excerpt of the *If I Were Jack* interactive film. Second, it includes education and guidance for parents in the form of two short animated films and a ‘JACK Factsheet’ to inform them about the homework activity, information about the importance of communicating with their child about teenage pregnancy and sexual health, and hints and tips for doing so.

**Fig. 1:**
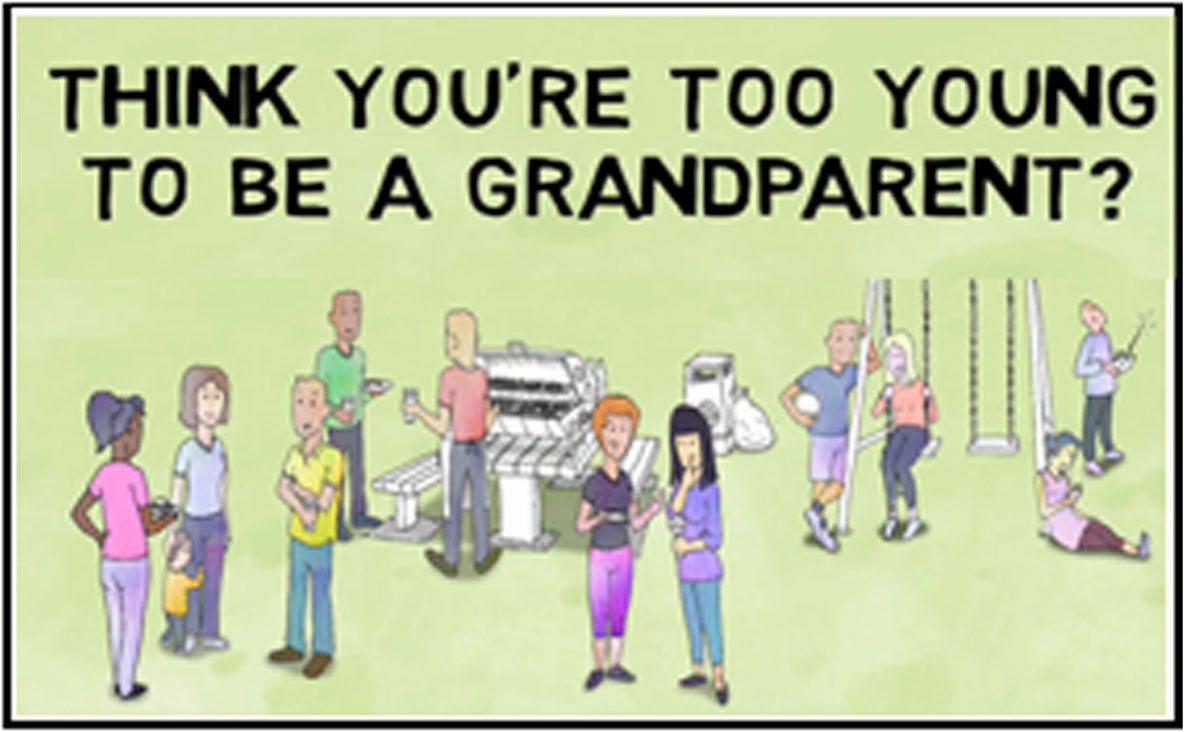
Hook Feature Screenshot

**Fig. 2:**
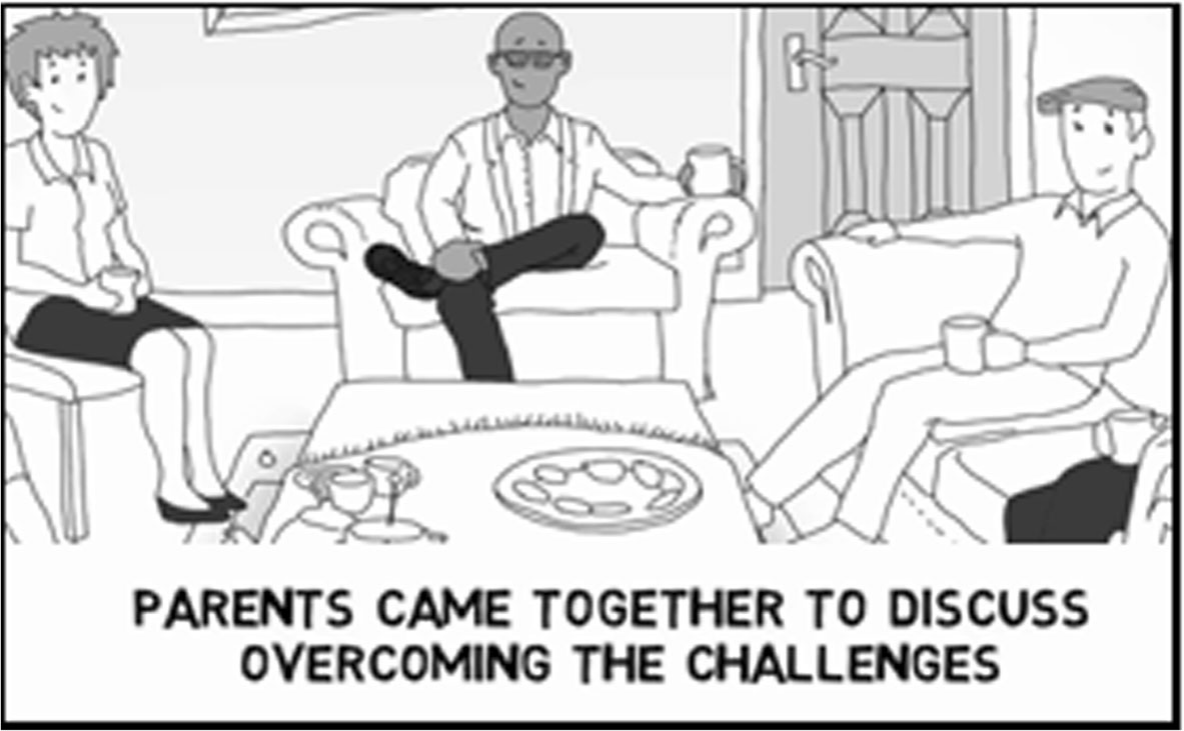
Instructional Feature Screenshot

The animated films were co-produced with separate groups of parent and expert stakeholders in 2016 and are designed to be viewed on mobile phones, tablets or computers. They include a 90-s ‘hook’ feature (Fig. 1) designed to engage parents by alerting them to the importance of having conversations with their teenagers about SRH, presenting abstract animated characters from multiple ethnic backgrounds with voiceover provided by BBC Radio presenter Kathy Clugston. The hook feature directs parents to a second 11-min ‘instructional’ feature (Fig. 2) which presents the experiences of a group of real parents as they discuss overcoming the challenges they face speaking to their teenagers about sex and pregnancy.

Schools were instructed to signpost this information to parents, sending them hard copies of the *If I Were Jack Factsheet* and a link via text message or email to the parents’ resources section of the project website.

### Sample and data sources

Data for this study was collected between January 2018 and August 2019 as part of a mixed-methods process evaluation embedded within the JACK cluster randomised trial. The JACK Trial was conducted with 8220 adolescents aged 14–16 in 66 schools (33 randomly assigned to the intervention group) across Northern Ireland (NI), Wales, Scotland and England. Eight schools from the intervention group (2 schools in each country) were randomly selected to act as ‘case study schools’ to take part in the process evaluation. Table 1 summarises the data sources and participant numbers presented in the current paper.

**Table 1:** Sample and Data Sources

Data source	Number participants
**Parents**	
Online Survey	134
Individual semi-structured interviews	10
Web Analytics (unique visits to parent resources section of website)	1123
**Students**	
Programme engagement and satisfaction questionnaire	3179
Focus groups (case study schools)	8 groups, *n* = 58
**Teachers**	
Focus groups (case study schools)	8 groups, *n* = 31
Implementation records	130
**RSE policy experts**	
Semi-structured interviews	10

### Study procedures

The intervention was delivered to approximately 4097 students (48% male) in the 33 intervention group schools by 175 teachers. A link to a short online survey relating to perceptions of the programme was texted or emailed by schools to the primary parent/carer contact of participating students (n = approximately 4097) and 3% of these were returned (*n* = 134, 2 male). Parent participants for the semi-structured interviews (*n* = 10, 1 male) were recruited via requests for volunteers from case study schools and respondents to the online survey. In the eight case study schools, teachers who delivered the programme (8 groups, *n* = 31) and adolescents (8 groups, *n* = 58) volunteered to take part in focus group discussions. In all intervention schools (*n* = 33), 3179 adolescents (47% male) completed a short student programme engagement and satisfaction questionnaire when implementation was complete. Other data sources included implementation records completed by teachers who delivered the programme (*n* = 130) and website viewing statistics for the implementation period obtained using Google Analytics.

All interviews and focus groups were audio recorded and transcribed anonymously. Schools were offered £1000 for participating in the trial, adolescents were provided with snacks during data collection sessions and parents who completed the survey or took part in interviews were entered into a prize draw for £500. Adolescents and parents were not offered incentives for participating in the programme. In order to counter possible contamination, the resources pages of the website were hidden and only accessible to those with a link to a specific page (i.e. parents could only access the parents’ resources using the link and the pages could not be accessed via the main website pages or using a Google search). Ethical approval for the study was provided by Queen’s University Belfast.

### Data analysis

Qualitative data were organised using NVivo 11 software. First, data were systematically analysed to provide evidence relating to the sub-components of user engagement (implementation fidelity, acceptability and feasibility). Second, data relating to general barriers and facilitators to parental communication were analysed thematically based on the six steps proposed by Braun and Clarke [81] to enable identification and analysis of patterns (or ‘themes’) within the data by moving iteratively between theoretical understandings and the new data. These inductively and deductively derived codes were first compiled as a code book and then applied to the data which was then analysed to form overarching themes emerging from each of the participant groups outlined above. Data from the parents’ survey and student satisfaction questionnaire were imported to Excel and SPSS and tabulated as summary statistics. For the programme engagement and satisfaction questionnaire, mean differences between groups were examined using Independent t-tests and Tukey’s Honest Significance tests. Qualitative responses to both questionnaires were analysed in the same way as the interview data. Table 2 illustrates which data sources were analysed for each of the concepts explored during the study.

**Table 2:** Data sources analysed for parental engagement and constituent concepts explored during the study

	Parental engagement			
	Implementation fidelity		Acceptability & feasibility	Barriers & facilitators
	Parent fidelity	Teacher fidelity		
**Data sources**	Parent surveys	Implementation logs	Parent surveys	Parent surveys
	Parent interviews	Teacher focus groups	Parent interviews	Parent interviews
	Student programme engagement and satisfaction surveys	Student programme engagement and satisfaction surveys	Student programme engagement and satisfaction surveys	Student programme engagement and satisfaction surveys
	Student focus groups	Student focus groups	Student focus groups	Student focus groups
	Website viewing statistics		Teacher focus groups	Teacher focus groups
				Expert interviews

## Results

Findings relating to parental engagement are reported below with reference to three sub-concepts: implementation fidelity; user perceptions of the acceptability and feasibility of the programme materials and exercises; and general barriers and facilitators to engaging parents in digital RSE.

### Implementation fidelity for digital materials

As highlighted in Table 3, 50% of 134 parent survey respondents, said they had watched the short animated film, while 45% had watched the longer animation. Of those who did not watch the films, the majority (68%) said it was because they did not know about them, 14% said they forgot to watch them, 11% said they did not have time and 4% said they did not interest them. Forty-two percent of parents said they watched the *If I Were Jack* interactive film excerpt.

**Table 3:** Parent responses to the online survey (*n* = 134, 3% response rate)

Survey item	Yes % (n)	No % (n)	Not sure % (n)
Parent watched shorter animated film for parents	50% (67)	50% (67)	–
Parent watched longer animated film for parents	45% (60)	55% (74)	–
Animations helped prepare parent to talk to teen	67% (48)	15% (11)	18% (13)
Parent watched *If I Were Jack* interactive film excerpt	42% (56)	58% (78)	–
Parent completed homework exercise with teen	34% (46)	55% (73)	11% (15)
Teen discussed experiences of using *If I Were Jack* with parent	55% (74)	45% (60)	–

Website analytics gave a broader view of parent fidelity, revealing a total of 1123 unique visits to the ‘parent resources’ section of the website during the implementation period. This indicates that approximately 27% of parents contacted (*n* = 4097) visited this section of the website on at least one occasion. Further, analytics showed that 380 (9%) viewed the shorter animated film, 288 (7%) viewed the longer animation, and 658 (16%) viewed the interactive film excerpt. Probably because parents who completed the survey were more likely to be engaged with the programme in general, these figures suggest lower rates of fidelity than reported by survey respondents.

During interviews, parents shared some suggestions for improving parent fidelity. In particular, they felt that compulsory homework exercises or more regular school contact with parents about RSE programmes might increase fidelity:

*Maybe more regular updates. You know, we had session one, and now we’ve had session two, just like a wee overview, you know, just like through email, because then you can say, “Oh, you had this today – tell me about that, you know, what was that part about”?* (Parent, NI).

Some mentioned how a mix of methods for involving parents might help with fidelity, with one parent suggesting that teacher facilitated face-to-face discussions between parents and young people might serve as a good ‘icebreaker’. Others were adamant that the approach of sending out information was likely to be much more useful to parents and some teachers suggested offering both:

*I think, instead of just a text message, giving them a chance to maybe come in and speak to us […] so you’ve definitely got that parental engagement then.* (Teacher, Wales)

### Implementation fidelity for parent-teen homework exercise

As noted, the digital parental materials were intended as a precursor to prepare parents for an optional parent-teen communication exercise assigned by schools. Teachers were instructed to assign the homework exercise to students, informing them that while the worksheets would not be collected, they should seek to ‘survey’ a parent/carer or other trusted adult using the worksheet. Across the UK, an implementation rate of 38% was reported by teachers for this, ranging from 29% implementation in Wales to 50% in England. Further, while 34% of parent survey respondents said they had completed the homework exercise with their child, student responses to the engagement questionnaire gave a broader view, revealing that only 13% of students (*n* = 403) said they had completed the task with their parents. Interestingly, student-reported figures varied by site, with 17% reporting completion in NI, 11% in England and Wales and 10% in Scotland. However, post hoc Tukey’s HSD revealed the only significant differences in country completion rates were between NI and the other three countries. Variations in completion rates according to gender were also evident with an independent t-test showing significantly more female pupils reporting completing the parental homework than males t(2955) = 2.5, *p* = 0.011. NI showed the starkest differences between females (24% completion) and males (9% completion) t(828) = 5.4, *p* < 0.01. Conversely, in England, 15% of males reported completion, compared with 10% of their female counterparts, which was also a significant difference in the other direction t(652) = − 2.12, *p* = 0.03. Differences between males and females in Scotland and Wales were less evident (no significant differences), with 10% of males compared with 11% of females in Scotland t(644) = .47, *p* = 0.64, and 13% of males compared with 12% of females in Wales reporting they completed the parent exercise with their parents t(825) = −.49, *p* = 0.65 .

Reasons given by teachers for non-delivery of the homework exercise were mostly student refusal and/or reluctance at a school or individual teacher level. In terms of student refusal, teachers said they either did not set the homework because their students felt uncomfortable doing it, or they did set homework but students did not complete it:

*When they had to go home and engage with their parent or guardian or other interested adult, they didn’t want to do that. So, I would say, out of a class of 30, perhaps five did that.* (Teacher, Wales)

*I think the majority of parents aren’t comfortable talking about these things, you know, with their kids, and when you speak to the kids, they say there is no way they would discuss this with their parents* (Teacher, Scotland)

Some schools decided to omit this activity from the outset. Anticipated controversy associated with RSE topics and parental engagement was cited as a main barrier to why some schools avoided parental inclusion:

*And one of the things we’re facing at the moment is the sort of post-Birmingham backlash. So, a number of our Muslim parents are writing me letters and are wanting their children withdrawn from RSE lessons.* (Teacher, England)

*You’re going to get a lot of kickback from a lot of the families in a school like ours that don’t want to talk about that sort of thing.* (Parent, England)

*The issues now are the same as they’ve been all along. There is a discomfort with the topic that some people have […] They’re afraid of the backlash and all those kind of things, or just because they’re uncomfortable personally talking about the issue.* (RSE Expert, Wales)

When asked in focus groups about reasons for not completing the exercise, the vast majority of young people said that it felt uncomfortable or ‘awkward’ to raise these issues with their parents, suggesting that it was better to allow these conversations to occur naturally:

*Like if I get homework, I do it obviously, but then, “Go home and talk to your parents about it”, and I was just a bit, yeah, “I’m not doing that.” I can’t go up to my mum and just mention it at the dinner table!* (Student, Scotland)

*If your parents are going to talk to you about that, they’re going to bring it up anyway and it will come in their own time. Like I…I’ve already had that talk and all about – so I don’t need the teacher being like, “Oh, so by the way, your homework is: go home and do this”, because then you’re just a bit like “Don’t want to!”* (Student, Wales).

Reflecting differences in socio-demographics in participating schools, teachers offered some possible reasons for lack of parent fidelity in relation to the homework exercise:

*For me, [the homework exercise] came across as being quite a middle-class activity, you know, the idea of going home and sitting around the table and talking about, “So, this is what we did at school, Mum, you know, can we talk about it?” because (a) a lot of our kids haven’t got tables, and (b) they’re certainly not going to talk about school and contraception and getting pregnant because, however ridiculous it might be, they just don’t do it.* (Teacher, Wales)

*I would say [sighing], sometimes the parents, not always, it’s not really fair to say that, but I think that they…they certainly prioritise [RSE] less because I think they think the reason kids are at this school is because it’s about academic achievement*. (Teacher, England)

### Acceptability and feasibility of digital parent materials

Of parent survey respondents who watched the animated films, 87% rated them as ‘good’ or ‘excellent’ and 67% said they helped prepare them to talk to their child about sex and pregnancy. During interviews, most parents shared very positive experiences, noting that they had found the animations ‘funny’ and ‘informative’.

*The wee one with the parents chatting. I thought that was quite funny and very real, you know, that we’re all in the same situation. We’re all a bit clueless at times.* (Parent, NI)

Most parents interviewed said they had already had conversations with their children about sex. However, some said that the animations had given them ideas about how to bring up what could be ‘embarrassing’ conversations, and others felt that they had motivated them to talk about relationships in more depth.

*They were all very good. […] The video helped me to start discussions with [my son], how to do it in a more casual way rather than a formal sit-down, so it’s not confrontational for them or embarrassing.* (Parent, Wales)

Many parents mentioned the fact that the programme had reminded them about the importance of speaking to their sons about these issues:

*It’s probably made me a wee bit more aware of keeping the channel of communication open, and more so with my son.* (Parent, NI)

Some parents said that they did not watch the animations because they felt they did not need further information and were satisfied with the conversations they had already had with their children or felt that sex was not something that their child was interested in yet.

*We’re quite fortunate, we’re still at the stage where it’s football and mates are the big thing. Girlies are there, but it’s not really [laughing] a big deal yet* (Parent, NI).

Others suggested changes that might improve acceptability of the digital materials including cutting the animations to a shorter length so they were more ‘short and punchy’ and thinking of a more hard-hitting ‘hook’ that would better grab parents’ attention. In particular, older parents mentioned that they were not in fact ‘too young to be a grandparent’:

*I’m not [too young to be a grandparent] because I didn’t have him till I was 35 [laughing], so that bit didn’t really relate to me* (Parent, NI).

### Acceptability and feasibility of the parent-teen communication exercise

Despite low rates of implementation for the homework activity, reports on how it went when actually carried out, were positive. Of the 134 parents who completed the survey, 55% said that their child had discussed their experiences of using *If I Were Jack* with them and of the 403 students (55% female) who said they completed the exercise, 67% percent ‘agreed’ or ‘strongly agreed’ that their parents had enjoyed it. Student-reported acceptability differed according to gender and country with 75% of females and 57% of males agreeing that their parents had enjoyed the activity and overall figures ranging from 75% agreement in NI to 47% agreement in England (students in Wales and Scotland reported 68 and 71% agreement respectively).

Some teachers reported unexpectedly positive responses from parents:

*Some of mine did do the homework […] I had nice comments back – you know, “My mum said, oh, she would just give them a hug and say it was alright – we’ll deal with it” So, they had discussed it.* (Teacher, Wales)

*We got nothing back, which was unusual, from parents [about the homework task], which, for us, as a school, means that the parents were happy.* (Teacher, Scotland)

Likewise, some parents felt the homework exercise was very useful:

*There was like a homework that we were suggested maybe to do and chat, I tried to bring it up that way. And he did open up, you know, and talk about it, and I was actually quite surprised by some of the information that he was able to give me […] It was good to have that opportunity, em, quite naturally, rather than just a sit-down and now we’re going to talk. So, I felt it made it easier.* (Parent, NI)

A small number of students who completed the engagement and satisfaction questionnaire (1%, *n* = 28) reported that the parent homework was the activity they liked the least. Of those who provided a reason for disliking or not completing it, most said it was because it was awkward or boring:

*I least liked the homework activity with my mum because it was very awkward trying to talk about it for me.* (Female student, NI)

*I didn't like the homework as it is really awkward to talk to your parents.* (Male student, Scotland)

*The parent/carer worksheet - it was quite boring and did not involve much discussing of interest to me.* (Male student, England)

Furthermore, some teachers highlighted that for many young people the homework exercise had a significantly negative impact on their engagement with the resource:

*And then they had to take it home and talk to their parents, and that was the point when everything changed. Because, until that point, they absolutely loved it. They were totally with me and loved it. When they then had to do things that they couldn’t do, the attitude changed completely.* (Teacher, Wales)

### Barriers and facilitators of parental engagement

While few participants directly suggested changes to the JACK programme materials, we identified a number of themes relating to barriers and facilitators of parental engagement in general that might serve as indicators for future programme development (see Table 4). Themes relating to barriers included: 1. fear about the political correctness of speaking to adolescents about sex or that talking about sex meant encouraging sexual activity; 2. religious beliefs and cultural norms that are not aligned with the provision of comprehensive RSE; 3. lack of knowledge about sexual health on the part of parents leading to a lack of confidence; and 4. lack of awareness among parents about the important role that parents play in relation to RSE. Themes relating to facilitators included: 1. Early and sustained intervention targeting parents of younger children when embarrassment was less likely and providing gradual directed support for parents who need it to continue conversations throughout childhood and adolescence; and 2. providing adequate SRH education for parents to increase awareness that RSE is important and a joint parent-school responsibility.

**Table 4:** Thematic summary of barriers to and facilitators of parental engagement, with illustrative extracts

Theme	Illustrative extracts
**Barriers**	
1. Fear about political correctness and condoning sexual activity	⇒ *You’re sort of wondering…should I? Do you know what I mean? You know, because everything nowadays is so PC that I think parents are even scared to chat about it, you know, in case, “Oh here, hold on a minute, why are you talking to me about sex?”* (Parent, NI) ⇒ *You say, […] “You’ve got a girlfriend, but you’re too young, so don’t do it.” You don’t then want to say, “But if you do…” because it’s almost condoning it […] I guess some people would just be under the impression that you’re just leading your children to be promiscuous*. (Parent, Wales) ⇒ *You know, certain people have really strong beliefs that we shouldn’t be teaching children about sex anyway because it promotes either homosexuality or promiscuity. Neither of those things are true.* (RSE expert 1, England)
2. Religious beliefs and cultural norms	⇒ *[My children’s father is] very much… “They grow up soon enough, there’s no point in discussing stuff like that.”* (Parent, Wales) ⇒ *There’s a girl I know, she’s Christian, and I said my daughter was doing the [JACK] trial and she nearly didn’t speak to me for a week! […] She doesn’t think it is appropriate that a 15-year-old should know all about those different things.* (Parent, NI) ⇒ *[My son] said that they were asked who was the most important person to tell or to talk about it with, […] and he was saying the mum or dad or the GP, eh, but the…a lot of his class all said it was the religious leader that they’d have to speak to.* (Parent, England) ⇒ *A lot of schools actually deliver RSE through RE, Religious Education, which isn’t the best place for it to sit because that has a moral perspective to it […] It becomes a moral right or wrong, whereas actually that’s not how sex kind of works.* (RSE Expert 1, England) ⇒ *Something I think that might be an issue is where the topic conflicts with the teacher’s own personal values. And it is very hard to say…to step back, particularly if it’s a very strong religious perspective on something or a very strong value about something.* (RSE Expert 2, NI) ⇒ Possibly reflecting their familial values, some students commented on how the intervention materials were not in line with their cultural or religious beliefs: ⇒ [On the JACK film] *‘Un-relatable, Imma Christian’*. (Male Pupil, NI) ⇒ [On the JACK film] *‘Haram’* [forbidden by Islamic law]. (Male Pupil, England) ⇒ [Least favourite activit*y] ‘The one about teen pregnancy because I have very strong feelings against abortion’.* (Female Pupil, Wales) ⇒ *[Least favourite activity] ‘Most of them because sex before marriage isn’t supposed to happen’.* (Female Pupil, England)
3. Parents’ lack of knowledge about sexual health	⇒ *Some parents need educated themselves, to be perfectly honest with you. I think some parents are in a different world. They just think that their child will never have sex [laughing] and they just have this idea that, you know, it’s never going to happen to their child.* (Parent, NI) ⇒ *And a lot of the time, the parents have had bad experiences of education, so the kids are actually more educated than the parents and [kids] don’t feel comfortable in talking about it because they feel that they’ve got to explain things to their parents that their teachers are explaining.* (Teacher, Wales) ⇒ *And that’s the big issue, is that the evidence doesn’t get to parents unless you can get them in a room and have a conversation with them. But schools aren’t doing that. The government isn’t doing that. The media is certainly not doing that.* (RSE expert 1, England)
4. Parents’ lack of awareness about the importance of their role	⇒ *And I think sometimes parents don’t know how to approach it…so let’s just ignore that. Let’s just pretend that wee letter didn’t come home from school. And the school will just do that anyway.* (Teacher, NI) ⇒ *I think it’s maybe trying to put an onus on the parents – “You are actually responsible for this part of your child’s health and wellbeing as well,” you know, it’s not up to school all the time either.* (Parent, NI) ⇒ *Over the last 20 years of trying to involve parents in these kinds of subjects and activities, I think I’ve only had one group that was really interested and successful. I’ve found it difficult, on a number of levels. My opinion would be to get in early.* (RSE expert, Wales)
5. Lack of RSE training and support for teachers and resulting lack of confidence	⇒ *There aren’t enough trained teachers out there, which then means there’s not enough confidence in delivering topics, particularly around the more sensitive issues in RSE.* (RSE expert 2, England) ⇒ *We need staff training for all teachers who deliver RSE, which basically means all staff. If we get good quality training for all staff, not just a few key teachers, then staff will be more comfortable teaching it.* (Teacher, NI) ⇒ *[Teachers might think] “I don’t feel comfortable with dealing with it, I haven’t had the training.” You know, sort of controversial issues, no matter what they are, are difficult. They don’t want to open a can of worms, they’re afraid. Or sometimes they’re afraid of what – they won’t get the backing from their own management. Or they’re maybe concerned that parents might complain.* (RSE Expert 2, NI) ⇒ *It’s always a fear of schools, raising [the issue of RSE] too much, in case you will get that parent that will say, “No, I don’t want them [to do it]* (Teacher, Scotland)
**Facilitators**	
**Theme**	**Illustrative extracts**
1. Early, sustained, gradual intervention	⇒ *It’s normalising those conversations. So, the analogy I use when I talk to parents, I say it’s like road safety. We talk to really small children all the time about road safety, and when you’re out with your child and you’re stood at the side of the road, you say, “Right, we need to look left, we need to look right”, and you practise those behaviours with your child because you know that one day they’re going to be crossing that road on their own. You don’t wait until they’re 10 or 11 and they’re going out on their own to teach them about it because it doesn’t work. You need to practise those behaviours.* (RSE expert 1, England) ⇒ *Maybe get them involved early on, so it’s not too late to get them talking to their kids.* (Parent, NI) ⇒ *We do see a difference in the children that are coming from [primary] schools that have a really good grounding [in RSE]. There’s no issue talking about RSE in post-primary then. (*RSE Expert 1, NI)
2. SRH education for parents and promoting RSE as a joint parent-school responsibility	⇒ *Sometimes parental concern is as much about the fact that they haven’t received this type of education in their own schooling and are worried that their children might have questions about things that they don’t know how to talk about. Some schools have taken the approach of doing things like having sex education libraries that are accessible to parents, so that parents can actually increase their knowledge and experience before young people have this education as well*. (RSE expert, England) ⇒ *Sometimes those can be very difficult conversations to initiate - you know, once they’re started, great, but I felt sometimes your child would maybe prefer to talk to someone that they’re not so close to, you know, about something like that as well. So, I really welcomed the fact that [the school] were trying to be proactive* (Parent, NI).

## Discussion

The parents who shared their experiences of using the digital JACK materials were very positive about them, with most rating them highly and describing them as useful for helping them to initiate and normalise conversations with their children; reminding them in particular of the importance of speaking about these issues with their sons. With participants suggesting only minor changes and additions to the materials, we can tentatively conclude that OMT-based SRH programmes for parents, such as the JACK digital materials, may represent an exemplar for future programme development. These findings and their possible implications should, however, be considered in the context of the broader information on user engagement uncovered during this study and discussed below.

### Implementation fidelity

Overall parent fidelity with the digital materials was moderate/low with just under a third of eligible parents accessing the website and less than a fifth viewing the animated films and interactive film excerpt. While these findings reflect challenges in involving parents in SRH education in schools reported in other research [69–71], it is difficult to draw direct comparisons with similar studies as few have used OMTs to improve parent-teen communication about SRH. As noted, we located only three comparable studies [76–78] and all report study retention rates rather than implementation fidelity making it difficult to know how many parents actually carried out the required elements of the intervention. Further, unlike the current study, all offered monetary incentives for implementation fidelity, thereby making it difficult to disentangle the efficacy of the programme (versus the incentive) for parent fidelity. When compared with non-OMT studies that did not offer incentives, which report fidelity rates of between 7 and 10% [52, 72], the current results are favourable. Considering this, and given the implications that using digital media for engaging parents might have for reach and reduction in resources required to deliver RSE, it could be argued that even moderate increases in fidelity rates, as demonstrated in the current study, suggest that digital methods are a promising means of increasing parental engagement with RSE. Importantly, the findings of this study suggest that potential ways of improving parent fidelity to digital exercises might involve increasing communication between schools and parents when programmes are ongoing and providing SRH information and support for parents at an earlier stage in their children’s lives. Further research is needed to examine the possible value of this approach.

A second, and vitally important, consideration in interpreting these findings on fidelity is that it is possible that more than half of eligible parents may not have received any or all information about the parent components from schools. Over a third of parents who completed the survey said they ‘did not know about’ the parent materials and, due in part to concerns that the exercise would be awkward for adolescents or result in backlash from parents, just over a third of teachers implemented the parent-teen homework exercise with fidelity. Further, when asked to confirm that they sent links about the online materials to parents, only half of participating schools could say for definite that they did so. These findings, reflecting those of previous studies [82, 83], suggest that implementation failure on the part of teachers may have obscured our ability to fully capture parental engagement in this instance. While it is likely that busyness and other priorities played a part in the failure of some schools to send out information to parents, the findings of this study suggest that these negative impacts on implementation fidelity might have been lessened had school management teams, including school secretaries and teachers, been provided with training and tools that would give them more confidence in engaging with parents. Programmes that seek to fill this gap are warranted.

A third consideration relates to the fact that the vast majority of parent participants were female. Although we specified in our recruitment materials that we were interested in recruiting both male and female parents/caregivers to the study, we recruited only one father to take part in interviews and only two of the parent survey respondents were male. Further, as we were unable to record the gender of parents who accessed the online materials and were unable to tell what percentage of the schools’ primary contacts (to whom the programme materials were sent) were male or female, we are limited in relation to conclusions we can draw about the feasibility of engaging fathers with OMTs of this nature. While it is important to be mindful that adolescents will not always have same-sex preferences in relation to parent communication about sex [54], given the important role that male caregivers might play in RSE [58, 84] and young men’s expressed desire for their fathers to provide them with information about sex [54], this is an important area for further research.

### Acceptability and feasibility of the JACK digital parent materials

While the majority of parents who used the digital materials were positive about them, it is important to note that some said they did not engage with the materials because they did not think they were relevant to them. These findings offer valuable information for future programme development. Echoing findings from other studies [43, 44, 85], central reasons for choosing not to use the materials were that they had already had conversations with their child about sex, they did not believe it was part of their role as a parent, or they believed their child was not yet interested in sex. While some parents will have frequent, open conversations with their child about sex, research also suggests that these are in the minority and while many parents report *they* are satisfied with the conversations they have had with their child, *their child* may not feel the same [86, 87]. Further, Mollborn et al. [44] found that 55% of parents surveyed guessed incorrectly about their child’s sexual experience and Pariera et al. [85] found that many parents were not aware of the value of speaking to their children about SRH. These findings highlight the importance of programmes that indicate the important role that parents play in SRH education and directly address the inconsistencies between parent and child experiences. Our study suggests that short and punchy sound-bites, films or animations addressing these key issues and co-produced with parents might offer an effective solution [75]. As noted, our hook feature ‘Think you’re too young to be a grandparent?’ was perceived by some parents as not hard-hitting or attention-grabbing enough and not relevant to them because they were, in fact, old enough to be grandparents. Further research with parents in this regard is warranted but alternative hooks might seek to target the underlying beliefs of such parents e.g. ‘Wish you knew how to talk to your child about sex? 'This approach is in line with research which suggests that parents who perceive that a programme may help address their children’s problems are more likely to engage [88–91].

In considering the optimisation of OMTs to improve communication, it is important to note that due to the small numbers of fathers who took part in the study, we are unable to draw conclusions in relation to the acceptability of OMTs for male caregivers or examine more closely the possible impact of gender differences on parent views on the acceptability of the digital materials. The very fact that we had difficulties recruiting fathers to the study might suggest that that gendered norms that place mothers as primary sex educators were in play. Echoing the findings from previous research [56, 57], the one father we were able to interview, expressed views that his wife was responsible for communication with their children about sex and related his fears that speaking about sex as a father was taboo. Future research might examine the acceptability and impact of programme materials designed specifically for male caregivers, paying particular attention to effects on communication with sons and daughters.

### Acceptability and feasibility of the JACK parent-teen communication exercise

Notwithstanding the potential for social influence or ‘groupthink’ effects in the context of focus group settings [92], this study also revealed that for the majority of adolescents the parent-teen communication component was not acceptable. Again this is difficult to compare with other studies as while most report on the impact of homework exercises for improving communication between parents and teens, they do not report the acceptability of the component to users or consider the possible implications of incentives for engagement in this regard [93, 94]. In the current study, some participants suggested that the structured nature of the exercise was problematic. Perceived adolescent expectations that it would be ‘awkward’ completing the activity with their parents were coupled with parent reports that while they had conversations with their children about the *JACK* programme, this generally did not involve the use of the provided ‘survey’ worksheet. Teachers corroborated adolescent reports of perceived embarrassment and two-thirds made the decision (at either an individual or school level) not to assign this exercise (even though it was intended to be ‘optional’ for students rather than for teachers). Teachers, adolescents and parents agreed that, for some, the issue was not about awkwardness but the reluctance to do ‘homework’ for any subject if it was not mandatory and others highlighted that the existing parent-teen relationship quality, including ethno-cultural norms and parental RSE knowledge levels, had implications for whether or not this was conducted. Relatedly, while exceptions were evident, many adolescents indicated that that they took a gendered approach when speaking to parents about sex, with girls preferring to speak to their mothers and boys to their fathers. Although we are limited in what we can conclude in this regard, due to the small number of male caregiver participants, it is possible that few fathers received or engaged with the parent materials, thereby limiting options for those adolescents who did have a preference for speaking to their male caregiver. Suggesting the possible implications of a lack of acceptability, some teachers indicated that the very mention of the exercise had completely changed students’ attitudes towards the programme. All this may highlight the possibility of the confounding effects of including parental RSE components such as this and future research should examine this possibility. It is a common but little considered implication that psychosocial learning interventions can cause iatrogenic effects [95–97], therefore, there is need to be cautious when implementing this type of programme. Further research examining the interactions between the components of such programmes, including that which is sensitive to gender differences in communication, is needed [74].

These findings suggest that a more flexible approach to stimulating conversations between parents and teenagers is needed. While some studies report success with games and videos to be watched by parents and teens together [34, 78, 98–100], another option, which is more in line with the findings of this study, is to allow parents and adolescents themselves to lead when and how these conversations happen. Indeed, the JACK parents’ animations teach parents to look for naturally occurring ‘teachable moments’ which they can use to speak to their children about sex and pregnancy, much in the same way that they talk about other health related issues [101]. Similarly, recognising that the needs of students and parents whose religious or cultural beliefs may not be in line with the provision of RSE in schools, programme developers should continue to work with key stakeholders to ensure the development of RSE materials that are acceptable to them and their communities [29]. In the current study, both teachers and parents highlighted their awareness of possible backlash from more religiously inclined parents but we noted no differences across faith- and non-faith based schools in terms of withdrawal of students from the programme or in reports of engagement with the parent materials. Although faith-based schools and religious parents are sometimes construed in mainstream media as opposed to any kind of RSE, research findings, including this study and our own previous work [3, 102], suggest that many are open to school-based RSE in general and most are open to the provision of RSE that is in line with their religious beliefs.

Parents and teachers in the current study suggested that attempting to have conversations *for the first time* during adolescence was always going to be challenging. In line with previous research [23], most parents who reported regular conversations about sex with their children said that these started early in childhood, usually prompted by the provision of age-appropriate RSE in their child’s primary school. With the current move towards providing age appropriate RSE to young children [47, 71], albeit in a context of controversy [46, 71, 103, 104] this holds promise for future generations in relation to the possibility of increased communication between parents and adolescents. Parents also noted that it was important that school input was sustained beyond primary school. Some mentioned that they would welcome more regular communication from schools about what their child was learning about in RSE, perhaps in the form of a short text message or email. Again this might provide them with ‘natural’ opportunities to start a conversation with their child, if the timing was right.

### Limitations of the study

The strengths of this study lie in its analysis of rich, contextually informed process data that uncovers user engagement with and perceptions of the acceptability of digital RSE materials for parents, demonstrating that, while work remains, it is possible to engage parents with such methods.

The study is not without its limitations, however, and in particular we note that engaging parents via schools was challenging. Some schools did not send parents the links to parental engagement materials and many students felt uncomfortable mentioning the homework assignment to parents, hence limiting their ability to engage with the materials. Related to this, we acknowledge that the response rate for the parents’ survey was low, and therefore subject to bias. We argue, however, that triangulation of parent survey findings with other sources of data including the web statistics, student engagement and satisfaction survey, and qualitative interviews increases the internal and external validity of the findings.

Another limitation of the study was that some groups were not adequately represented. As noted, fathers and male caregivers were not adequately represented in the study and this prevents us drawing conclusions about gender differences relating to acceptability and feasibility. Additionally, ethnic minorities, who make up a significant minority (around 17%) of the UK population, were not adequately represented in the co-production of the resource materials. Although initial study information sheets and parents’ factsheet were translated into four different languages, the animated films and parents survey were provided in English only. Given that many participating parents would have come from ethnic minority backgrounds it is possible that some (including those in Welsh speaking areas) did not engage with the digital materials because they were not provided in their language of choice. For these reasons, and given our understanding of the importance of ethnicity as a determinant of parent-teen communication, about sex [29], we suggest that further research examining this is vital.

## Conclusion

While it is recognised that both home and school play key roles in SRH education [37], there is a persistent problem with the fact that most existing school-based SRH programmes require a significant time commitment from parents, thereby limiting reach [26, 50]. Findings from this study indicate that while OMTs for parents are a promising avenue for future research and programme development, it is difficult to deliver digital parental SRH components with fidelity.

A key message for SRH programme developers and evaluators wishing to engage parents is this: If we are to encourage parents to engage with school-delivered digital RSE we need also to ensure that schools and teachers are willing and able to reach out to them. Ultimately, teachers need to feel it is appropriate for them to engage parents, and confident that they have the tools to enable them to do so successfully. Much could be achieved in this regard with the provision of high quality training and materials that would support schools and teachers to engage parents. Also, this study suggests that there is a need to engage parents with RSE consistently and gradually, so that capacity can be built between parents, children and appropriate teaching support. Crucially, this engagement process should be underpinned with the key message that parents and schools have complementary and important roles to play in promoting positive SRH; while schools have a role in providing comprehensive evidence-based RSE, parents have a role in sharing their own experiences, values, beliefs and expectations in relation to sexual behaviour and SRH.

Evaluation practitioners have a key role to play in insuring the continued availability of digital materials that are acceptable to parents. Going forward we recommend co-development of user-friendly programmes for particular groups including parents from socio-economically deprived backgrounds, ethnic minorities and fathers. The latter should pay particular attention to research which indicates the structural challenges that fathers face in relation to their roles as sex educators and be cognisant of the fact that they will be preferred sources of information about sex by some, but not all, adolescents.

Further research is also needed to ensure that faith-based schools and schools with religiously diverse populations are adequately supported to provide comprehensive RSE in a way that is in line with their ethos and acceptable to parents, while also respecting the child’s right to age-appropriate RSE. Equally, a continued focus on gender-transformative programmes that encourage parents to have conversations with their sons as well as their daughters in order to help challenge gender inequalities relating to SRH is needed. A central message that emerged from this study is the need to bring parents and schools together to co-produce SRH materials for parents and provide school staff with training and materials that would increase their confidence and reassure them of the value of communicating with parents, even on sensitive topics.

## Data Availability

All relevant data are presented in the paper. Data will be archived in a UK data archiving depository when the project is completed. Intervention materials are embargoed until the completion of the JACK Trial in December 2020 after which they will be available on the project website www.qub.ac.uk/if-i-were-jack

## Abbreviations

NI: Northern Ireland
RSE: Relationships and Sexuality Education
SRH: Sexual and Reproductive Health
